# BAT3 Regulates *Mycobacterium tuberculosis* Protein ESAT-6-Mediated Apoptosis of Macrophages

**DOI:** 10.1371/journal.pone.0040836

**Published:** 2012-07-13

**Authors:** Ajay Grover, Angelo A. Izzo

**Affiliations:** Departments of Microbiology, Immunology and Pathology, Colorado State University, Fort Collins, Colorado, United States of America; Institut Pasteur, France

## Abstract

HLA-B-associated transcript 3 (BAT3), also known as Scythe or BAG6, is a nuclear protein implicated in the control of apoptosis and natural killer (NK) cell-dendritic cell (DC) interaction. We demonstrate that BAT3 modulates the immune response by regulating the function of macrophages. BAT3 is released by macrophages in vitro and it down-regulates nitric oxide and proinflammatory cytokines release in IFN-γ and LPS stimulated macrophages. Furthermore, Mycobacterium tuberculosis-derived protein ESAT-6 (Rv3875) induced transient increase in the expression and release of BAT3 in macrophages. We show that induction of apoptosis by ESAT-6 is dependent on the cleavage of BAT3 by caspase-3 and proteasomal degradation. Our results also indicate that BAT3 regulates ESAT-6-induced apoptosis by interacting with anti-apoptotic protein BCL-2. Taken together, the data suggest that BAT3 plays a role in the early immune response to M. tuberculosis infection and may be a key protein associated with the fate of antigen presenting cells during infection.

## Introduction

HLA-B-associated transcript 3 (BAT3) is a nuclear protein expressed by a gene located within the cluster of genes of major histocompatibility complex class III region (MHC class III), in the vicinity of genes for TNF-alpha and TNF-beta. BAT3 is structurally characterized by C-terminal nuclear localization signals, an N-terminal ubiquitin-like region, a polyproline stretch, and the conserved BAG (Bcl-associated anthogene) domain [1], [2]. BAT3 has been reported to regulate several functions of cell signaling. These include regulation of mammalian development, proteasome-based degradation of proteins, cellular proliferation and apoptosis. Nuclear BAT3 is responsible for the p53-mediated cellular response to stress and DNA damage, resulting either in DNA repair or in apoptosis, which ultimately suppresses tumor growth [3].

BAT3 is involved in the regulation of development and reproduction of mammals by acting as a co-chaperone of the heat shock protein HSP70 [4]. Its deficiency induces polyubiquitylation and subsequent degradation of HSP70 [5]. BAT3 is required for the accumulation of HSP70 upon heat shock; once accumulated, HSP70 leads to the degradation of BAT3 via a ubiquitin-proteasome mechanism. BAT3 also acts as a novel tethering factor that mediates selective elimination of defective nascent chain polypeptides in mammalian cells through ubiquitin-mediated degradation [6]. Some studies have highlighted the role of BAT3 in controlling the gene expression and cell division [7], [8]. For example, BAT3 is known to interact with histone H3 methyltransferase (SET1A), and exerts its effects upon chromatin structure and gene expression [7]. BAT3 also interacts with human small glutamine-rich TPR-containing protein (hSGT) and could be directly or indirectly required for complete chromosome congression during cell division [8].

Several studies have shown that BAT3 acts as a novel regulator of apoptosis that may regulate apoptotic pathways by interacting with other major proteins involved in the process. The invertebrate homologue of BAT3, known as Scythe, regulates apoptotic pathways during development [9]. Scythe regulates elongation factor XEF1AO-induced apoptosis during the course of Xenopus development and reaper-induced apoptosis in Drosophila development [10]–[12]. Scythe also physically interacts with apoptosis inducing factor and regulates its stability and is involved in endoplasmic reticulum (ER) stress-induced apoptosis [13]. In mammalian cells, the ribosomal inactivating protein ricin interacts with BAT3 and the complex binds to caspase-3, leading to cleavage of BAT3 and causing morphological changes observed in apoptosis [14]. BAT3 negatively regulates programmed cell death caused by papillomavirus binding factor in human osteosarcoma [15]. Taken together, these data suggest that BAT3 is implicated in programmed cell death during developmental processes and ER stress-induced apoptosis.

Little is known about the function of BAT3 in the immune response against cancer and infectious pathogens. BAT3 acts as a TGF-β receptor-interacting protein in kidney cells and regulates TGF-β signaling [16]. BAT3 is released by tumor cells, binds directly to natural killer (NK) cell receptor NKp30 and triggers NKp30-mediated killing of target cells [17]. BAT3 is released by immature dendritic cells (DC) and involved in NK-DC cross-talk, leading to NK cell activation [18]. In this study, we investigate the role of BAT3 in modulating the function of macrophages and then in relation to Mycobacterium tuberculosis infection. Our data show that BAT3 down-regulates the activation of LPS and IFN-γ stimulated macrophages.

*Mycobacterium tuberculosis* infection causes the induction of the apoptotic response, which is associated with bacilli killing. The immunodominant M. tuberculosis antigen ESAT-6 (early secreted antigenic target-6) is a small (6 kDa) protein has been shown to induce apoptosis in macrophages and epithelial cells [19], [20]. The secretion of ESAT-6 is required for *M. tuberculosis* virulence and pathogenicity [21]. ESAT-6 is one of the important targets for cell-mediated immunity in the early phase of tuberculosis (TB). Thus, ESAT-6 has been widely evaluated as a vaccine candidate and diagnostic tool [21]–[24]. We observed that ESAT-6 up-regulates BAT3 expression and may play a significant role in the pathogenesis of the disease. Furthermore, our results uncovered a novel regulation of the apoptotic mechanism of ESAT- 6 through interaction of BAT3 and BCL-2 in such pathologic conditions as TB.

## Results

### BAT3 is Released from Macrophages in vitro

**Figure 1:**
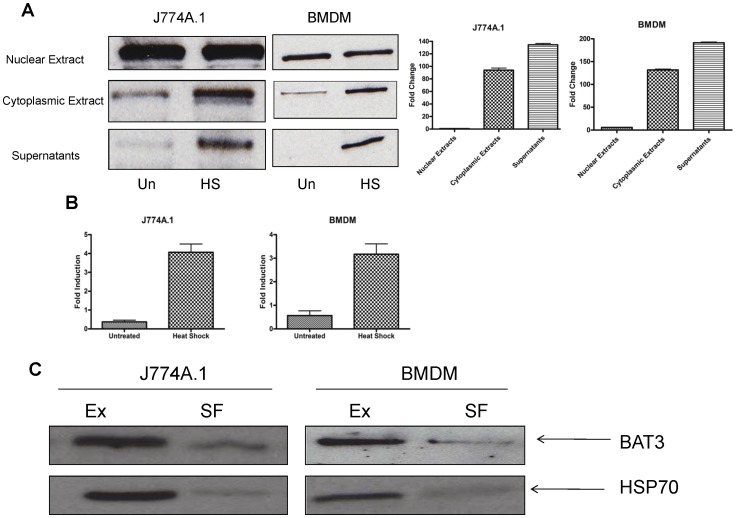
Expression of BAT3 in J774A.1 cells and bone marrow-derived macrophages (BMDM) in response to non-lethal heat shock. A. J774A.1 cells and BMDM were subjected to non-lethal heat shock at 42°C for 10 minutes and rested for 1 hour. Total protein concentration of different fractions was determined and equal amounts of proteins (25 µg) were loaded on SDS-PAGE gels. Following electrophoresis and western blotting, the blots were developed using a rabbit polyclonal antibody against BAT3 protein. The molecular weight of BAT3 was ∼122 kDa. Histogram plot shows the densitometric quantification of BAT3 protein levels shown in western blots. B. Total RNA was isolated from cells; cDNA was prepared and subjected to real-time PCR for BAT3 gene amplification. ΔΔCT values were normalized to mouse GAPDH gene. C. The exosomal pellet (Ex) and soluble fractions (SF), of both J774A.1 cells and bone marrow-derived macrophages (BMDM) culture supernatants were collected. Total 25 µg of protein from each sample was run on a 12% SDS-PAGE gel followed by western blotting to detect BAT3 (upper panel) and HSP70 (lower panel). The cytoplasmic marker GAPDH and nuclear marker histone H1 served as positive controls for the cytoplasmic extracts and nuclear extracts, respectively, in western blotting experiments.

To determine if BAT3 is released from J774A.1 murine macrophages and mouse bone marrow-derived macrophages (BMDM), cells were cultured in vitro and subjected to non-lethal heat shock. The expression of BAT3 was detected in the nucleus, cytoplasm and supernatants of macrophages under normal culture conditions. Under non-lethal heat shock, BAT3 expression increased in the cytoplasm and cell supernatants (Figure 1A). The cytoplasmic marker GAPDH and nuclear marker histone H1 served as positive controls for the cytoplasmic extracts and nuclear extracts, respectively, in western blotting experiments (data not shown). An increase in BAT3 expression was also observed in the cytoplasm and cell supernatants of bone marrow-derived dendritic cells (BMDC) and DC2.4 dendritic cell line in response to non-lethal heat shock (Figure S1A). Heat shock elements were present in BAT3 promoter at position −125 and within the first intron of the ubiquitin-like domain of BAT3 [25], suggesting that heat shock may control BAT3 expression during transcription. Therefore, we investigated the regulation of BAT3 expression at the transcription level using real-time PCR. An increase in BAT3 mRNA levels was observed in J774A.1 and BMDM cells in response to non-lethal heat shock (Figure 1B). A similar increase in BAT3 mRNA levels was observed in BMDC and DC2.4 cells (Figure S1B). These results support that of others who have shown that BAT3 is released from human tumor cells [17] and immature DCs [18] in response to non-lethal heat shock. It is known that immature dendritic cells and 293T cells release BAT3 in secreted exosomes [18]. We observed that BAT3 is present not only in exosomes secreted by J774A.1 and BMDM, but also in the soluble fraction. As shown in Figure 1C, most of the BAT3 protein was present in exosomal fractions, but some BAT3 was also present in soluble fraction. The exosomal marker HSP70 served as a positive control for western blot analysis.

### Soluble BAT3 Modulates Macrophage Function

**Figure 2:**
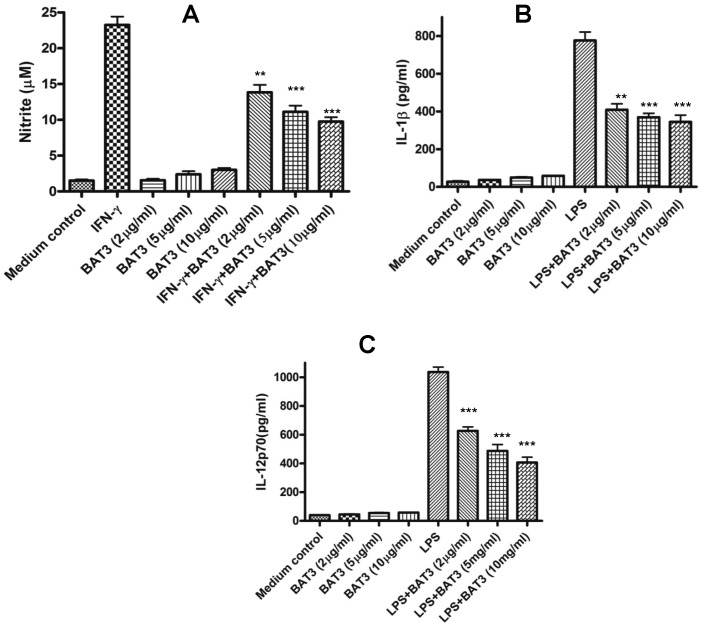
Modulation of macrophage functions by soluble BAT3. A. J774A.1 cells were first stimulated with IFN-γ (20 ng/ml) for 2 hours and then purified recombinant BAT3 was added into culture at different concentrations. Total nitrite levels in cell culture supernatants were determined after 18 hours using a colorimetric assay kit. **P<0.01 and ***P<0.001 as compared with IFN-γ only group. B. J774A.1 cells were first incubated with LPS (100 ng/ml) for 2 hours and then stimulated with BAT3 at different concentrations. The levels of IL-1β in cell culture supernatants were determined after 24 hours using ELISA kit from eBioscience Inc. **P<0.01 and ***P<0.001 as compared with LPS only group. C. J774A.1 cells were incubated with LPS (100 ng/ml) for 2 hours and then stimulated with BAT3 at different concentrations. The levels of IL-12p70 in cell culture supernatants were determined after 24 hours using ELISA kit from eBioscience Inc. ***P<0.001 as compared with LPS only group.

The immunological function of exosomal BAT3 has been characterized earlier [17], [18]. To investigate the effects of soluble BAT3 in regulating macrophage function, J774A.1 cells were stimulated initially with recombinant murine IFN-γ (20 ng/ml) for 2 hours and then with purified recombinant BAT3 for 18 hours. IFN-γ-induced nitric oxide production in macrophages was significantly down-regulated by BAT3 (p<0.001) in a dose-dependent manner (Figure 2A). J774A.1 cells were then incubated with LPS (100 ng/ml) for 2 hours, treated with BAT3, and levels of IL-1β and IL-12p70 were assessed after 24 hours. BAT3 significantly down-regulated LPS stimulated IL-1β (p<0.001) and IL-12p70 (p<0.001) (Figure 2B & C) production by J774A.1 cells. These results suggest that soluble BAT3 down-regulates macrophage activation. In contrast, when BAT3 was over-expressed in macrophages, the levels of TNF-α, IL-1β and IL-12 were not significantly altered (data not shown). In order to determine if BAT3 had a regulatory effect on DC activation, murine BMDCs were first stimulated with LPS (100 ng/ml) for 2 hours and then treated with recombinant BAT3 (5 µg/ml). The cells were analyzed for the expression of DC activation markers CD80, MHC class II and CD86 by flow cytometry after 48 hours. As reported by others [26], LPS enhanced the expression of CD80 on the surface of CD11c^+^ DCs. The up-regulation of CD80 was reduced in the presence of recombinant BAT3 (Figure S2A). Similar results were seen for MHC class II (Figure S2B) and CD86 expression (Figure S2C).

### ESAT-6 Protein (Rv3875) of M. tuberculosis Induces the Expression of BAT3 in Macrophages

**Figure 3:**
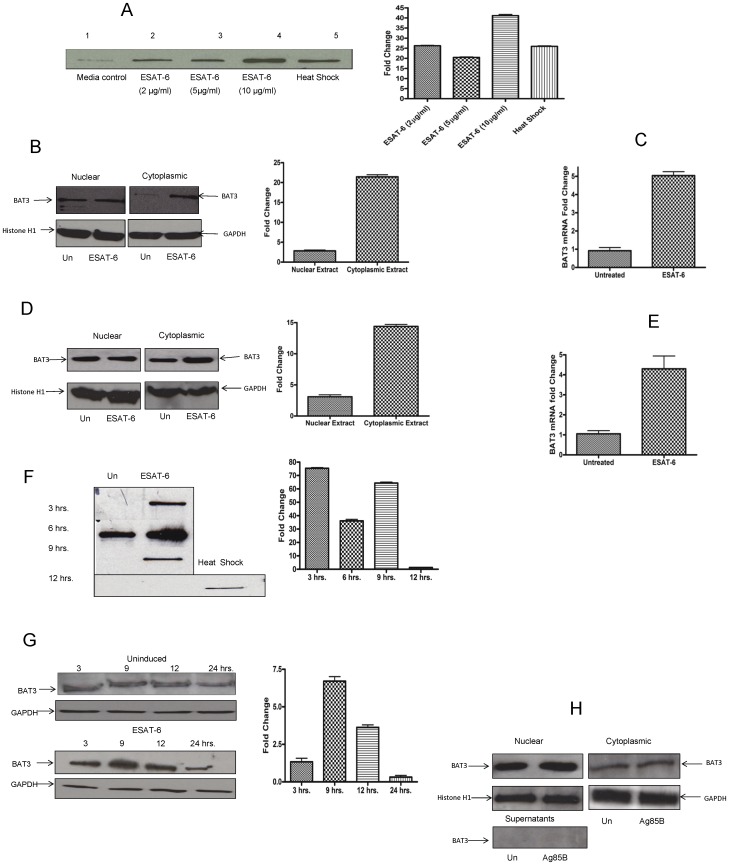
ESAT-6 induces the expression of BAT3 in macrophages. A. Bone marrow-derived macrophages (BMDM) were incubated with different concentrations of ESAT-6 in Opti-MEM medium for 6 hours. Cell culture supernatants were collected and concentrated. Total protein concentration of different fractions was determined and equal amounts of proteins (25 µg) were loaded on an SDS-PAGE gel. Following electrophoresis and western blotting, the blot was developed using a rabbit polyclonal antibody against BAT3 protein. Histogram plot shows the densitometric quantification of BAT3 protein levels shown in western blot. B. BMDM were incubated with 5 µg/ml of ESAT-6. Nuclear and cytoplasmic extracts were prepared and western blots were developed as mentioned above in figure 3A. Histogram plot shows the densitometric quantification of the change in BAT3 protein levels in nuclear and cytoplasmic extracts. C. Total RNA was isolated from BMDM that were stimulated with ESAT-6 (5 µg/ml) for 6 hours; cDNA was prepared and subjected to real-time PCR for BAT3 gene amplification. ΔΔCT values were normalized to mouse GAPDH gene. D. J774A.1 cells were incubated with 5 µg/ml of ESAT-6 and western blots of nuclear and cytoplasmic fractions were developed as mentioned above in figure 3A. Histogram plot shows the densitometric quantification of the change in BAT3 protein levels in nuclear and cytoplasmic extracts. E. J774A.1 cells were stimulated with ESAT-6 (5 µg/ml) for 6 hours. Total RNA was isolated; cDNA was prepared and subjected to real-time PCR for BAT3 gene amplification. ΔΔCT values were normalized to mouse GAPDH gene. F. J774A.1 cells were incubated with 5 µg/ml of ESAT-6 and culture supernatants were collected at different time points. Heat shock-treated cell supernatants were used as positive control. Western blots were developed as mentioned above in figure 3A. Histogram plot shows the densitometric quantification of BAT3 protein levels shown in western blot. G. J774A.1 cells were incubated with 5 µg/ml of ESAT-6 and cytoplasmic extracts were collected at different time points. The western blots were developed as mentioned above in figure 3A. Histogram plot shows the densitometric quantification of BAT3 protein levels shown in western blot. H. J774A.1 cells were incubated with 5 µg/ml of Ag85B for 9 hours and different cellular fractions (Nuclear extract, Cytoplasmic extract and Supernatants) were collected at different time points. The western blots were developed as mentioned above in figure 3A. The cytoplasmic marker GAPDH and nuclear marker histone H1 served as positive controls for the cytoplasmic extracts and nuclear extracts, respectively, in western blotting experiments.

To investigate the role of BAT3 in the modulation of macrophage response after infection with intracellular pathogens such as M. tuberculosis, we examined the effect of a major immunodominant protein ESAT-6 on the expression of BAT3. Because M. tuberculosis resides within macrophages and ESAT-6 is produced early during the infection process, we postulated that it may have an effect on macrophage function. Previously, we showed that the expression of high mobility group box 1 (HMGB1), a nuclear danger signal protein in macrophages, was modulated by ESAT-6 [27]. Here, we report that expression of BAT3 and its release by macrophages is increased in vitro in response to ESAT-6 protein. When incubated with BMDM, ESAT-6 induced the release of BAT3 into the extracellular environment (Figure 3A). An increase in expression of BAT3 protein was observed in cytoplasmic extracts (Figure 3B) and BAT3 mRNA (Figure 3C) in response to ESAT-6. Similar data was obtained when J774A.1 cells were stimulated with ESAT-6 in vitro for BAT3 protein (Figure 3D) and mRNA (Figure 3E). The extracellular release of BAT3 in the same cultures was observed during the initial hours following stimulation with ESAT-6 and no BAT3 protein was detected in cell culture supernatants after 9 hours (Figure 3F). These results indicate that ESAT-6 induces enhanced expression and release of BAT3. Because the extracellular release of BAT3 in macrophages was observed in the first few hours following ESAT-6 stimulation, we monitored the expression of BAT3 in the cytoplasm of J774A.1 cells at different time intervals after incubation with ESAT-6. The increase in expression of BAT3 was found to be transient, with the highest expression observed at 9 hours following ESAT-6 stimulation (Figure 3G). BAT3 levels in the cytoplasm returned to normal at 24 hours. To assess the specificity of the induction in BAT3 expression, we evaluated another *M. tuberculosis* recombinant protein, Ag85B for its ability to induce BAT3 expression in J7744A.1 cells. When incubated with J774A.1 cells, Ag85B did not induce the expression of BAT3 in cytoplasmic extracts and extracellular environment (Figure 3H).

### Regulation of ESAT-6 Induced Apoptosis by Intracellular BAT3

**Figure 4:**
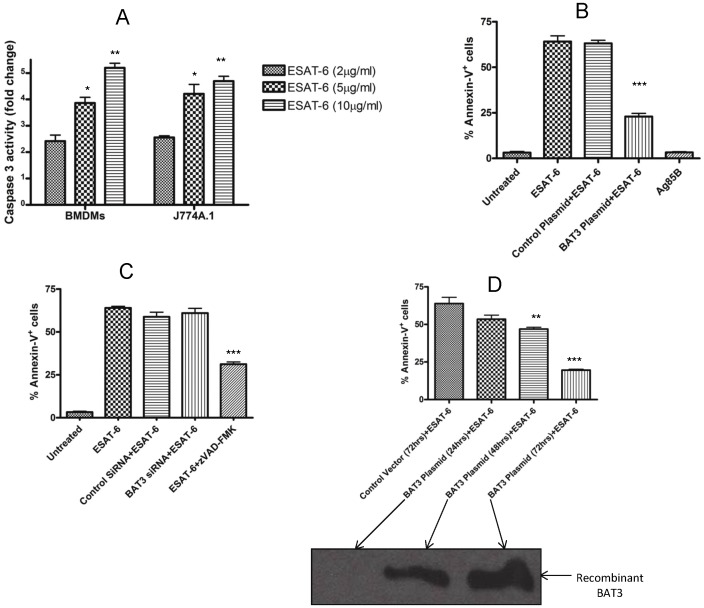
Regulation of ESAT-6 induced apoptosis by BAT3. A. BMDMs and J774A.1 cells were incubated with different concentrations of ESAT-6 for 24 hours. Cell culture supernatants were collected and subjected to colorimetric caspase-3 assay. Fold change in comparison to unstimulated controls was calculated and plotted in bar graph. *P<0.1 and **P<0.01 as compared with ESAT-6 (2 µg/ml) group. B. J774A.1 cells were transfected with BAT3 plasmid or control vector and then stimulated with 5 µg/ml of ESAT-6 protein for 24 hours. The cells were stained with PE labelled AnnexinV and 7-AAD and subjected to flow cytometry for determination of Annexin V positive and 7-AAD negative apoptotic cells. Percentages of apoptotic cells were plotted in a bar graph. J774A.1 cells treated with 5 µg/ml of Ag85B protein served as negative control for apoptosis. ***P<0.001 as compared with control plasmid+ESAT-6 group. C. J774A.1 cells were transfected with BAT3 siRNA or control siRNA or pre-incubated with 85 µM zVAD-FMK for 4 hours and then stimulated with 5 µg/ml of ESAT-6 protein for 24 hours. The cells were stained and subjected to flow cytometry to determine apoptosis as mentioned above in figure 4B. ***P<0.001 as compared with ESAT-6 only group. D. J774A.1 cells were transfected with control vector for 72 hours or BAT3 plasmid for time intervals ranging from 24 hours to 72 hours and then stimulated with 5 µg/ml of ESAT-6 protein for 24 hours. The cells were stained and subjected to flow cytometry to determine apoptosis as mentioned above in figure 4B. **P<0.01 and ***P<0.001 as compared with control vector (72 hours) +ESAT-6 group. Lower panel shows the western blot of recombinant BAT3 expressed in the cytoplasmic extracts of BAT3 plasmid transfected cells obtained at different time intervals. Total 25 µg of each protein sample was loaded in 12% SDS-PAGE gel for the development of western blot.

ESAT-6 protein is known to activate caspases and induce apoptosis in THP-1 macrophages, DC2.4 cells and human epithelial cells [19], [20]. We observed that ESAT-6 also activates caspase-3 in BMDMs and J774A.1 macrophages within 24 hours (Figure 4A), with the response dependent on ESAT-6 concentration. ESAT-6-mediated apoptosis involves endoplasmic reticulum (ER) stress response and is dependent on extrinsic and intrinsic pathways [20]. ER stress is induced in macrophages of tuberculosis granulomas in areas where apoptotic cells accumulate in lungs of mice and humans [28]. Because BAT3 is involved in ER stress-related apoptosis [13], and its expression in macrophages is transiently induced by ESAT-6, we explored the role of BAT3 in ESAT-6-induced apoptosis. We over-expressed full-length recombinant BAT3 protein in J774A.1 cells, stimulated the cells with ESAT-6 and determined the percentage of Annexin-V positive cells using flow cytometry. The over-expression of BAT3 significantly reduced the number of Annexin V positive cells (p<0.001), indicating that BAT3 down-regulates ESAT-6-mediated apoptotic cell death (Figure 4B). No apoptotic cell death was observed in control vector transfected cells and cells incubated with Ag85B, another major secretory protein of M. tuberculosis. Next, we treated the J774A.1 cells with BAT3 siRNA and measured the levels of apoptosis. No change was detected in the percentage of Annexin V positive cells when BAT3 was knocked down in ESAT-6 treated J774A.1 (Figure 4C). However, pre-incubation of the ESAT-6 treated cells with caspase-3 inhibitor zVAD-FMK significantly reduced the apoptosis levels (p<0.01) compared to cells incubated with ESAT-6 alone. We then measured the apoptosis levels in cells over-expressing recombinant BAT3 at different time intervals after incubation with ESAT-6 protein. As observed in Figure 4D, the percentage of Annexin V+ cells was significantly lower at 48 hrs (p<0.01) and 72 hrs (p<0.001) in BAT3 plasmid-transfected cells as compared to untransfected cells. The highest levels of expression of recombinant BAT3 were found at 72 hrs in the cytoplasmic extracts of J774A.1 cells following transfection with BAT3 plasmid (Figure 4D). These results combined suggest that BAT3 transiently down-regulates ESAT-6-induced apoptosis. ESAT-6 induces an initial transient increase in the expression of BAT3 in the cytoplasm that prevents the cell entering in apoptosis pathway, but the concentration of BAT3 returns to basal levels due possibly to its degradation.

### BAT3 Interacts with BCL-2

**Figure 5:**
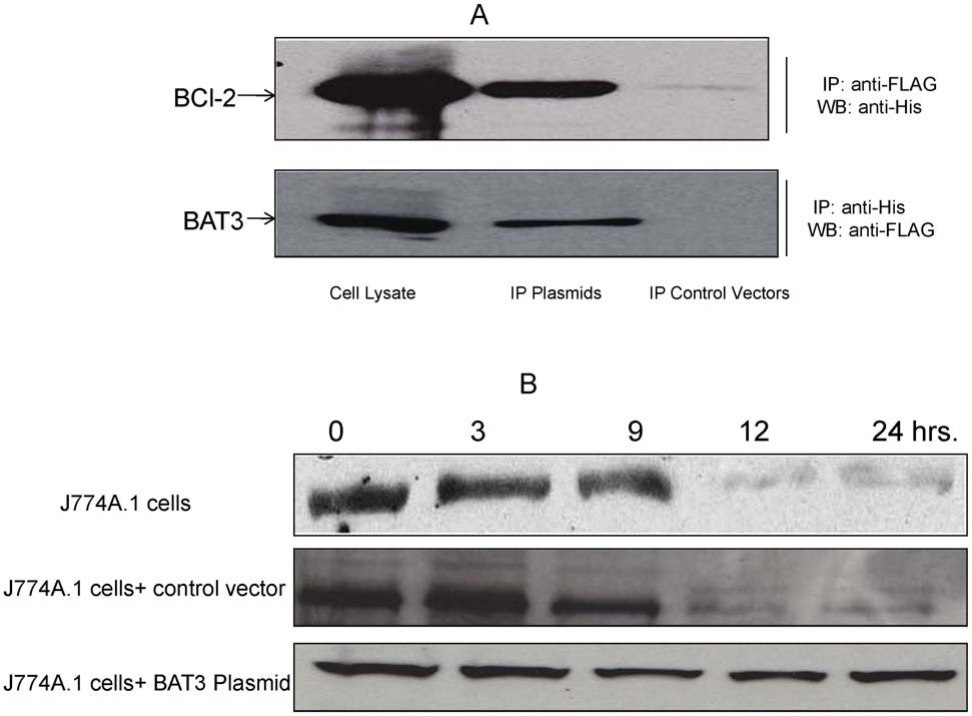
Interaction of BAT3 with BCL-2 and expression of BCL-2 in ESAT-6 stimulated J774A.1 cells. A. J774A.1 cells were co-transfected with FLAG-tagged BAT3 plasmid and HIS-tagged BCL-2 plasmid or control vectors and incubated with ESAT-6. Cytoplasmic extracts of the cells were subjected to immunoprecipitation (IP) with antibodies against FLAG peptide or HIS tag, followed by western blotting with respective antibodies as indicated. Total 25 µg of each protein sample was loaded in 12% SDS-PAGE gel for the development of western blot. Upper panel: IP with anti-FLAG antibody and western blot with anti-HIS antibody. The molecular weight of BCL-2 was ∼25 kDa. Lower panel: IP with anti-HIS antibody and western blot with anti-FLAG antibody. The molecular weight of BAT3 was ∼122 kDa. Whole cell lysates of the J774A.1 cells expressing recombinant BAT3 and BCL-2 proteins served as positive control for the western blotting. B. Upper panel: J774A.1 cells were incubated with 5 µg/ml of ESAT-6 and cytoplasmic extracts were collected at different time points. Total protein concentration of different fractions was determined and equal amounts of proteins (25 µg) were loaded on an SDS-PAGE gel. Following electrophoresis and western blotting, the blot was developed using a mouse monoclonal antibody against BCL-2 protein. J774A.1 cells were either transfected with BAT3 plasmid (lower panel) or control vector (middle panel) for 72 hours, incubated with ESAT-6 and cytoplasmic extracts were collected at different time points. Western blots were developed for the BCL-2 protein as described earlier.

The apoptotic cell death induced by proteins such as ricin and papilloma virus binding factor (PBF) is down-regulated by BAT3 via the mechanism of direct interaction of BAT3 with these proteins [14], [15]. This interaction leads to cleavage of BAT3 that further activates casapase-3 and leads to apoptosis. Because no such direct interaction was observed between ESAT-6 and BAT3 in the current study (data not shown), we considered that BAT3 may interact with other anti-apoptotic proteins when its expression level is high in the cytoplasm. BAT3 belongs to the BAG family of proteins that have an evolutionarily conserved BAG domain. Other members of the BAG family, such as BAG1 and BAG3, associate with anti-apoptotic protein BCL-2 (B cell lymphoma-2) through the BAG domain [29], [30]. We reasoned that BAT3 may also bind with BCL-2. J774A.1 cells were co-transfected with a FLAG-tagged BAT3 plasmid and His–tagged BCL-2 plasmid. Cytoplasmic extracts of the cells, subjected to immunoprecipitation (IP) using anti-FLAG tag antibodies, were analyzed by subsequent western blotting with anti-His antibody (Figure 5A upper panel); or, conversely, IP using anti-His antibody and western blotting with anti-FLAG antibody (Figure 5A lower panel). As shown in Figure 5A, recombinant BCL-2 and BAT3 proteins were detected in pull-down assays of the cytoplasmic extracts of cells co-transfected with BAT3 and BCL-2 plasmids, but not in pull-down assays of cytoplasmic extracts of cells co-transfected with the control vectors. Whole cell lysates of the J774A.1 cells expressing recombinant BAT3 and BCL-2 proteins served as a positive control for the western blotting. These results revealed that BAT3 interacts with BCL-2. We next analyzed the expression of BCL-2 protein in J774A.1 cells following stimulation with ESAT-6. The levels of BCL-2 decreased in the cytoplasm of J774A.1 cells after ESAT-6 treatment (Figure 5B top panel). Others have reported similar results in A549 epithelial cells [27]. We then studied the expression of BCL-2 in J774A.1 cells over-expressing recombinant BAT3 protein. J774A.1 cells transfected with BAT3 plasmid showed little or no change in the expression of BCL-2 protein after incubation with ESAT-6 (Figure 5B lower panel), but BCL-2 expression was decreased in the cells transfected with the control vector (Figure 5B middle panel). We found no change in the expression of BAT3 when recombinant BCL-2 protein was over-expressed in the J774A.1 cells after incubation with ESAT-6 (data not shown). These results demonstrate that the increase in expression of BAT3 stabilizes the BCL-2 protein.

### BAT3 is Degraded by Caspase-3 Cleavage and Proteasome

**Figure 6:**
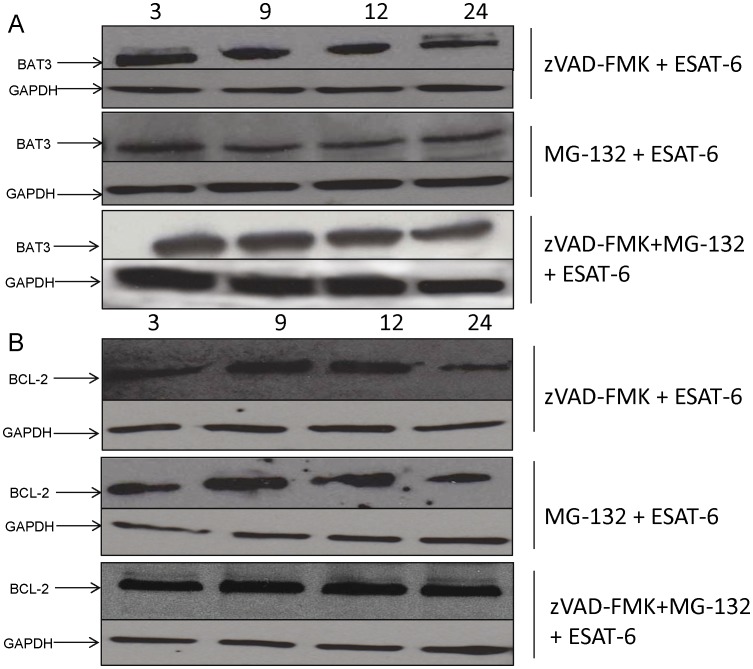
Inhibition of caspase-3 and proteasome provides protection of BAT3 and BCL-2. J774A.1 cells were pre-incubated with 85 µM zVAD-FMK or 10 µM proteasome inhibitor MG-132 or both for 4 hours and then stimulated with 5 µg/ml of ESAT-6 protein. Cytoplasmic extracts were collected at different time points. Total protein concentration of different fractions was determined and equal amounts of proteins (25 µg) were loaded on an SDS-PAGE gel. Following electrophoresis and western blotting, the blots were developed using a rabbit polyclonal antibody against BAT3 protein (Figure 6A) and a mouse monoclonal antibody against BCL-2 protein (Figure 6B). All experiments were done with 0.05% DMSO vehicle control and ESAT-6 alone as positive control (data not shown). The cytoplasmic marker GAPDH served as positive control for the western blots of cytoplasmic extracts.

Some members of the BAG family of proteins are known to have putative caspase-3 cleavage sites [14], [31] and act as a substrate for caspase-3. BAT3 (also called BAG6) has a canonical caspase-3 cleavage site, DEQD^1001^, and acts as a substrate of caspase-3 during ER stress induced apoptosis [14]. The loss of BAT3 upon activation of the intrinsic apoptosis pathway indicates that BAT3 may be a target of caspase-3 activated by the ESAT-6-induced extrinsic pathway of apoptosis. Therefore, we treated the J774A.1 cells with zVAD-FMK (predominantly caspase-3 and -1 inhibitor) and then stimulated the cells with ESAT-6. The presence of caspase-3 inhibitor prevented the loss of BAT3 in cytoplasmic extracts of cells in the presence of ESAT-6 (Figure 6A upper panel). But, inhibition of caspase-3 only partially protected the loss of BAT3, indicating that additional pathways may be needed for complete loss of BAT3. The anti-apoptotic activity of BAG3, another protein of the BAG family, is restricted by caspases and proteasomal degradation [31]. Since caspase-3 controls the loss of BAT3 in ESAT-6-induced apoptosis, we considered that proteasomal degradation could be another mechanism responsible for degradation of BAT3. Reversible proteasomal inhibitor MG132 was found to prevent degradation of BAT3 when J774A.1 cells were incubated with ESAT-6 (Figure 6A middle panel). We next investigated the complementarity of zVAD-FMK and MG132 on BAT3 rescue. No degradation of BAT3 was seen when both inhibitors were used together (Figure 6A lower panel). These data suggest that the primary means of degrading BAT3 is via caspase-3 cleavage, but proteasomal degradation is also responsible for the degradation of BAT3 in ESAT-6-induced apoptosis. We next analyzed if inhibition of caspase-3 and proteasome degradation prevented the loss of BCL-2 since BAT3 stabilizes BCL-2 during ESAT-6-induced apoptosis. We observed that the incubation with caspase-3 inhibitor (Figure 6B upper panel) and MG-132 (Figure 6B middle panel) also prevented the degradation of BCL-2 to some extent when J774A.1 cells were incubated with ESAT-6. The addition of both inhibitors resulted in near complete BCL-2 retention in the cytoplasmic extracts of J774A.1 cells during ESAT-6-induced apoptosis (Figure 6B lower panel). Overall, our observations indicate a key role for the interaction of BAT3 and BCL-2 in ESAT-6-induced apoptosis and suggest that interlinked extrinsic and intrinsic pathways lead to the degradation of BAT3.

## Discussion

A growing body of evidence indicates that nuclear proteins may modulate the functions of immune cells in cancer, apoptosis and infection, but little is known about the functions of nuclear protein BAT3 in immune cells. Recent studies have shown that BAT3 released by human DCs in culture, acts as an important ligand of NK cells and is involved in NK cell-mediated cytotoxicity [17], [18]. We observed that the expression of BAT3 was increased in cytoplasm of murine macrophages in response to non-lethal heat shock and ESAT-6 protein of M. tuberculosis. BAT3 was also released by these cells in culture supernatant. This phenomenon was not associated with apoptosis or necrotic cell death (data not shown). BAT3 is therefore accessible to interact with immune cells.

The data presented here show that BAT3 may behave similarly to a nuclear immune regulatory factor, such as HMGB1, that is released by DCs and macrophages in response to endotoxin shock and infection, as reviewed elsewhere [32]. But, the functions of BAT3 observed in vitro differed from those of HMGB1. We could not identify any interaction of BAT3 with TLRs or RAGE, known receptors of HMGB1. Soluble BAT3 down-regulated the production of IFN-γ-mediated nitric oxide and LPS-induced cytokine release in J774A.1 cells. BAT3 is known to interact with TGF-β receptor and act as positive regulator of the TGF-β signaling pathways in mammalian cells [16]. We postulated that the modulation of IFN-γ and LPS-mediated functions of BAT3 in J774A.1 cells could be attributed to this interaction since TGF- β also down-regulates IFN-γ-stimulated nitric oxide release [33] and LPS-mediated proinflammatory cytokine release [34], [35]. However, we found that the interaction of TGF-β receptor was not involved in these functions (data not shown). Further studies are needed to determine the mechanism responsible for the immune modulation functions of BAT3. BAT3 had an inhibitory effect on LPS-induced activation of BMDCs as revealed by down-regulation of CD80, CD86 and MHC class II markers. Others have also observed that the soluble form of BAT3 down-regulates activation of immature DCs in NK cell- mediated DC-maturation [18]. Taken together, we demonstrated that the soluble form of BAT3 has inhibitory effects on immune functions of macrophages and dendritic cells.

Our previous studies of the nuclear protein HMGB1 revealed that proteins secreted by mycobacterium in culture induced release of HMGB1 into the extracellular environment [27]. In this study, we showed that ESAT-6, a major secretory protein of *M. tuberculosis* induced a transient increase in the expression and release of BAT3. Because BAT3 was found to down-regulate ESAT-6-induced apoptosis, we propose that high levels of BAT3 induced by ESAT-6 in the cytoplasm initially resist apoptotic changes in the cell, but BAT3 then degrades due to caspase-3 cleavage and proteasomal degradation. Another member of the BAG family of proteins, BAG3, is also degraded by caspases and proteasomal degradation during ER stress-induced apoptosis [31]. The transient increase in expression of BAT3 and its release by macrophages peaked at 3 hours of ESAT-6 stimulation and returned to basal levels after 9 hours. ESAT-6-induced apoptosis of macrophages has been observed only after 16 hours of stimulation in culture [19]. The transient increase in expression of BAG3 has been observed in a similar way during ER stress-driven apoptosis [31].

Both the mechanisms and functional consequences of the role of BAT3 in apoptosis have been the subject of intense investigation and debate. Initial studies done on Scythe, an invertebrate homologue of BAT3, revealed that it positively regulates cell death in mammalian development [9] and thapsigargin-induced apoptosis by controlling the stability of apoptosis inducing factor (AIF) [13]. Other studies have shown that BAT3 is an anti-apoptotic protein and negatively regulates cell death caused by reaper [12], ricin [14] and papilloma virus binding factor (PBF) [15]. Several mechanisms have been proposed for negative regulation of cell death by BAT3. BAT3 was proposed to be normally bound to a pro-apoptotic factor which is then released when reaper interacts with BAT3 in reaper-induced apoptosis [12]. Ricin binds to BAT3 and causes caspase-3 based cleavage of BAT3, releasing a C-terminal fragment that causes characteristic changes of apoptotic cell death [14]. PBF interacts with BAT3 in the nucleus and brings transcriptional changes that lead to PBF-induced cell death in osteosarcoma [15]. Our study has found neither an interaction of BAT3 with ESAT-6 nor release of C-terminal fragment in ESAT-6-induced apoptosis (data not shown). We observed that BAT3 interacts with BCL-2, an anti-apoptotic protein involved in the intrinsic pathway of apoptosis and this interaction negatively regulates ESAT-6-induced apoptosis. In addition, BCL-2 was degraded when J774A.1 cells were incubated with ESAT-6. Similarly, BCL-2 was degraded during ESAT-6-mediated apoptosis of epithelial cells [20]. We showed that BAT3 controls the ESAT-6-induced apoptosis by stabilizing BCL-2, but this phenomenon occurs only when ESAT-6 induces a transient increase in the expression of BAT3. The high levels of BAT3 in the cytoplasm may provide access for the interaction with BCL-2 that is mostly localized in the ER and mitochondrial membranes. Our data suggest that BAT3 degradation may start primarily by the activation of caspase-3 induced by the extrinsic pathway, followed by proteasomal degradation. This phenomenon may further activate an intrinsic pathway through the loss of BCL-2. However, a constant level of BAT3 is maintained in the nucleus and cytoplasm of the cell that does not initiate apoptotic changes in a cell. This is consistent with our findings that the over-expression of full-length BAT3 in macrophages reduced apoptosis, but apoptotic cell death was not affected by knocking down BAT3 expression (Figure 5A & B). Together these data suggest that BAT3 functions as a pro-survival protein in tuberculosis, with ESAT-6 inducing its expression, and is regulated by caspase cleavage and proteasomal degradation. In summary, our data describes a novel regulatory complex between BAT3 and BCL-2, thereby providing new insights into the basic physiology of macrophage cell death in tuberculosis.

## Materials and Methods

### Cells, Animals and Other Reagents

J774A.1 murine macrophage cell line (ATCC # TIB-67), 293T human embryonic kidney cell line (ATCC # CRL-11268) and DC2.4, an immortalized mature dendritic cell line [36], were cultured in DMEM or RPMI media, supplemented with 10% FBS, 10 mM HEPES, 2 mM L-glutamine, 100 U penicillin per ml, 100 µg streptomycin per ml, and nonessential amino acids. Recombinant ESAT-6 protein was provided by the TB Vaccine Testing and Research Materials Contract at Colorado State University (NIH, NIAID Contract No. HHSN266200400091C). Primers used in the cloning and real-time PCR of BAT3 were designed with Oligoperfect Designer software (Life Technologies, NY) and are described in Table S1. BAT3 polyclonal antibody was provided by Drs. Peter J McKinnon and Helen Russell of St Jude Children's Research Hospital, Memphis, TN. To obtain bone marrow-derived macrophages (BMDM) or bone marrow-derived dendritic cells (BMDC), femurs from C57BL/6 mice (The Charles River Laboratory, MA) were dissected free of connective tissue and flushed with DMEM medium. Bone marrow cells were seeded at 1 × 10^6^ cells ml^−1^ in the presence of 30% L929 conditioned medium for BMDMs or 20 ng/ml Granulocyte-macrophage colony-stimulating factor (GM-CSF) for BMDCs. On day 7 of culture, non-adherent cells were removed by vigorous washing with DMEM medium. All experimental protocols used in this study were specifically approved by the Animal Care and Use Committee of Colorado State University. Unless otherwise indicated, all experiments were repeated three times.

### BAT3 and BCL-2 Mammalian Expression Constructs

Total RNA was extracted from J774A.1 cells using Trizol (Invitrogen, CA) according to manufacturer’s instructions. cDNA was produced by using an iScript cDNA synthesis kit (Bio-Rad). Full-length BAT3 and BCL-2 genes were amplified by polymerase chain reaction (PCR) using cDNA as template and were cloned in pFLAG-CMV-4 vector (Sigma) and pcDNA3.1/His vector (Invitrogen), respectively.

### Cell Culture

J774A.1 cells, BMDMs or BMDCs were plated at 3 × 10^6^ cells/well in 6-well culture plates and then stimulated with recombinant ESAT-6, recombinant BAT3, Lipopolysaccharide (LPS), Antigen 85-B and inhibitors such as zVAD-FMK or MG-132 or DMSO at indicated concentrations. Supernatants were collected for detection of cytokines, BAT3, nitric oxide and caspase-3 in different experiments. As required, cells were treated with non-lethal heat shock at 42°C for 10 minutes and rested for 1 hour. Nuclear and cytoplasmic extracts of the cells were prepared using CeLytic NuCLEAR Extraction Kit (Sigma) according to manufacturer's instructions.

### Biochemical and Immunochemical Assays

The levels of IL-1β and IL-12p70 in supernatants were measured from cultured J774A.1 cells using Ready-Set-Go ELISA kits (eBioscience), and assays were performed in accordance with manufacturer's instructions. For western blotting of BAT3 or BCL-2, supernatants of cultured cells, nuclear and cytoplasmic extracts were concentrated using Amicon ultra-4 centrifugal filter (Millipore). Protein concentration of concentrated supernatants, nuclear extracts and cytoplasmic extracts was determined by the BCA method (Pierce BCA kit) to ensure equal loading, and 25 µg of each sample was resolved on 12% Tris-SDS polyacrylamide gel. Protein was transferred to nitrocellular membranes, blocked with 5% bovine serum albumin in PBS-Tween, and blotted with primary antibodies, as indicated. The membrane was then incubated with HRP-conjugated anti-mouse IgG or anti-rabbit IgG (Jacksonimmuno Research), and developed using ECL chemiluminiscence kit (Pierce). The relative levels of proteins were quantified using Quantity One software (Bio-rad) after capturing Western blot images by a charge-coupled device camera. Caspase-3 assay was performed using caspase-3 colorimetric assay kit (Biovision) according to manufacturer's instructions. Briefly, ESAT-6- stimulated were lysed and the protein concentration in cell lysates was determined. The protein samples were incubated in a buffer containing DTT and DEVD-*p*NA substrate at 37°C for 1 hour. The absorbance was read at 405 nm and the fold increase in Caspase-3 activity was determined by comparing with the absorbance level of the uninduced control. The levels of nitric oxide in the supernatants of cultured cells were determined using Nitric Oxide Colorimetric Assay kit (Biovision). Quantitative real-time PCR was performed for amplification of BAT3 gene using iQ Syber green Supermix (Bio-Rad) after preparing cDNA from RNA isolated from cultured BMDMs, BMDCs, J774A.1 and DC2.4 cells.

### Expression and Purification of Recombinant BAT3

293T cells were transfected with BAT3 mammalian expression construct using Nanojuice transfection kit (EMD Biosciences). Western blotting was performed to confirm the expression of recombinant protein. BAT3 was purified from cell lysates using Anti-FLAG M2 affinity gel (Sigma) according to manufacturer’s protocol.

### Isolation and Purification of Exosomes

Exosomes were purified as described elsewhere [18]. In brief, exosomes were purified from the supernatant by three successive centrifugations at 300× g (5 min), 1200× g (20 min) and 10 000×g (30 min) to eliminate cells and debris, followed by centrifugation for 1 hour at 100 000×g. The exosomal pellet was washed once in a large volume of PBS, centrifuged at 100 000×g for 1 hour and re-suspended in PBS.

### Co-immunoprecipitation

The mammalian expression constructs pFLAG-CMV-4/BAT3 and pcDNA3.1/His/BCL-2 were transfected in J774A.1 cells. At 48–72 hours post transfection, the cells were washed with cold PBS and lysed using CelLytic M Cell Lysis Reagent (Sigma). Protein concentrations of the cell lysates were determined by the BCA method (Bio-Rad) and immunoprecipitation was carried out with 200 µg of protein lysates using Anti-FLAG M2 affinity gel (Sigma) and Ni-charged HisBind Resin (EMD chemicals) according to manufacturer’s instructions. Eluted samples were subjected to SDS-PAGE on a 12% gel and further processed for western transfer onto the nitrocellulose membrane. The membranes were blocked and blotted with anti-penta his (Qiagen) and anti-FLAG antibody (Sigma) separately. Further, they were incubated with the respective secondary antibodies conjugated to horse-radish peroxidase and subjected to standard chemiluminiscence (ECL kit, Pierce).

### Flow Cytometry and Apoptosis Assay

BMDCs were stained with fluorescence-labeled MAbs against CD11c, CD80, CD86 and MHC Class II (BD Biosciences, CA) at 4°C for 30 minutes in the dark, after the cells were washed with phosphate-buffered saline containing 0.1% sodium azide (Sigma-Aldrich). Antibodies were used at 0.2µg/10^6^ cells. Cells were gated on dendritic cells by forward and side scatter, according to their characteristic scatter profile and further gated based on CD11c and CD80 or CD86 or MHC class II expression. All analyses were performed with an acquisition of at least 100,000 events on a FACSCalibur flow cytometer (BD Biosciences), and the data were analyzed using CellQuest software (BD Biosciences, San Jose, CA). Annexin V staining of the cultured macrophages was performed to determine levels of apoptosis using PE Annexin V Apoptosis Detection Kit (BD Biosciences) according to manufacturer’s protocol. Flow cytometry analyses of apoptotic cells were performed on a Becton Dickinson FACSCalibur flow cytometer and data analyzed as mentioned above. Percentages of apoptotic cells were calculated by determining Annexin V positive and 7-AAD negative cells out of total number of cells.

### Gene Silencing

J774A.1 cells were transfected with Mm_BAT3_5HP SiRNA (Qiagen) or All Stars Negative Control SiRNA (Qiagen) using HiPerFect Transfection Reagent (Qiagen) as per manufacturer’s instructions. The levels of BAT3 protein in the cell lysates and cytoplasmic extracts were determined by western blotting using BAT3 polyclonal antibody as described earlier.

### Statistical Analysis

Two-way comparison between test and control group was performed using Student's *t*-test. The data are given as means ± standard error of the mean.

## Supporting Information

Figure S1 **Expression of BAT3 in bone marrow-derived dendritic cells (BMDC) and DC2.4 cells in response to non-lethal heat shock.** A. Western blots showing expression of BAT3 in nuclear, cytoplasmic and supernatant fractions of BMDC and DC2.4 cells. B. Total RNA was isolated from cells; cDNA was prepared and subjected to real-time PCR for BAT3 gene amplification. ΔΔCT values were normalized to mouse GAPDH gene.
(TIF)

Figure S2 **Effects of soluble BAT3 on expression of activation markers on dendritic cells.** Murine bone marrow-derived dendritic cells (BMDC) were first stimulated with LPS (100 ng/ml) for 2 hours and then treated with BAT3 (5 µg/ml). The cells were analyzed by flow cytometry for the expression of DC activation markers CD80 (A), MHC class II (B) and CD86 (C) after 48 hours. The cells were gated on CD11c and DC activation markers and dot plots are shown.
(TIF)

Table S1 Sequences of the primers used in Real-Time PCR of BAT3 and Molecular Cloning of BAT3 gene.
(DOCX)
