# Supplementary figures and images for: BAT3 Regulates Mycobacterium tuberculosis Protein ESAT-6-Mediated Apoptosis of Macrophages
Source: PLoS One. 2012 Jul 13;7(7):e40836. doi: 10.1371/journal.pone.0040836 (PMC3396635; doi:10.1371/journal.pone.0040836)

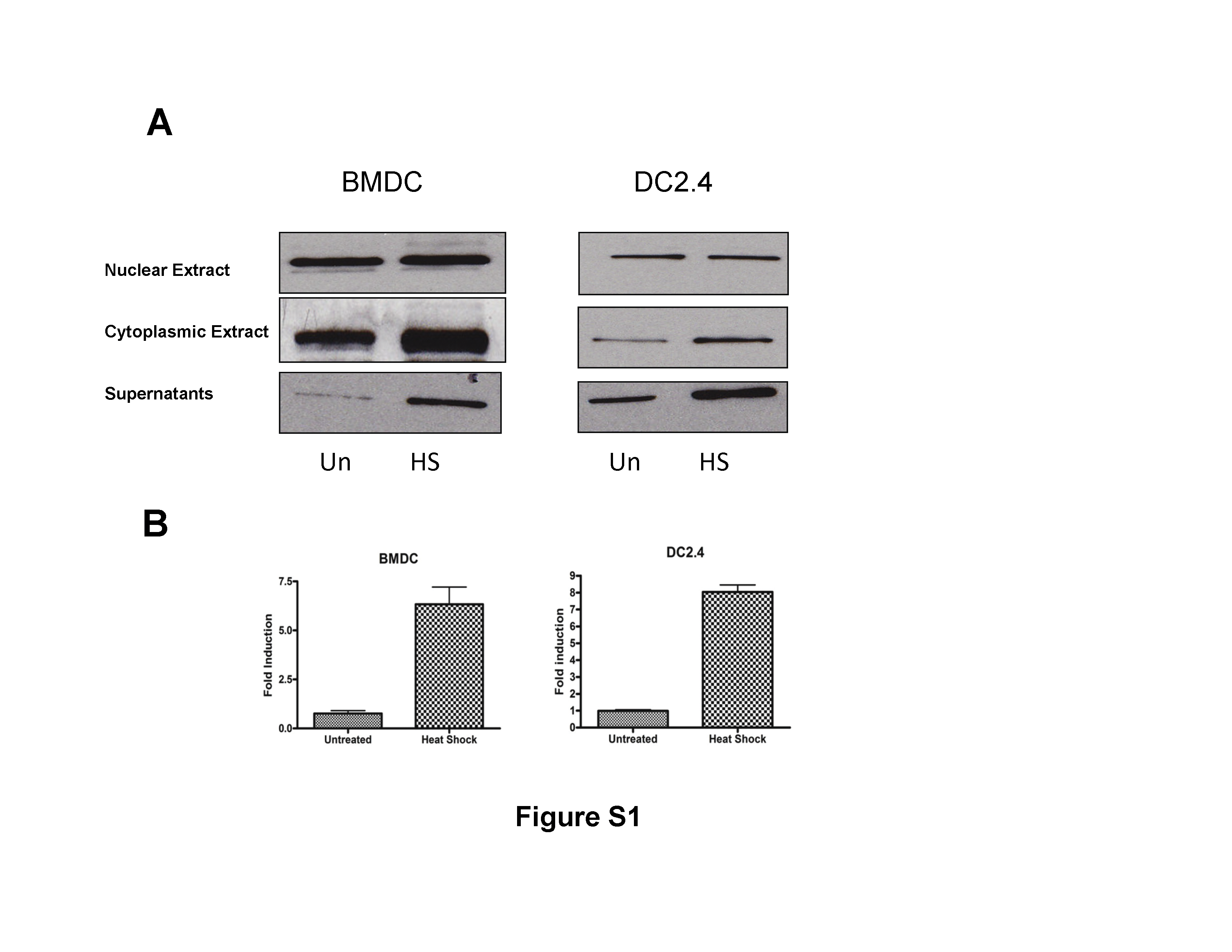

Supplement: Figure S1 — Expression of BAT3 in bone marrow-derived dendritic cells (BMDC) and DC2.4 cells in response to non-lethal heat shock. A. Western blots showing expression of BAT3 in nuclear, cytoplasmic and supernatant fractions of BMDC and DC2.4 cells. B. Total RNA was isolated from cells; cDNA was prepared and subjected to real-time PCR for BAT3 gene amplification. ΔΔCT values were normalized to mouse GAPDH gene. (TIF) [file pone.0040836.s001.tif]

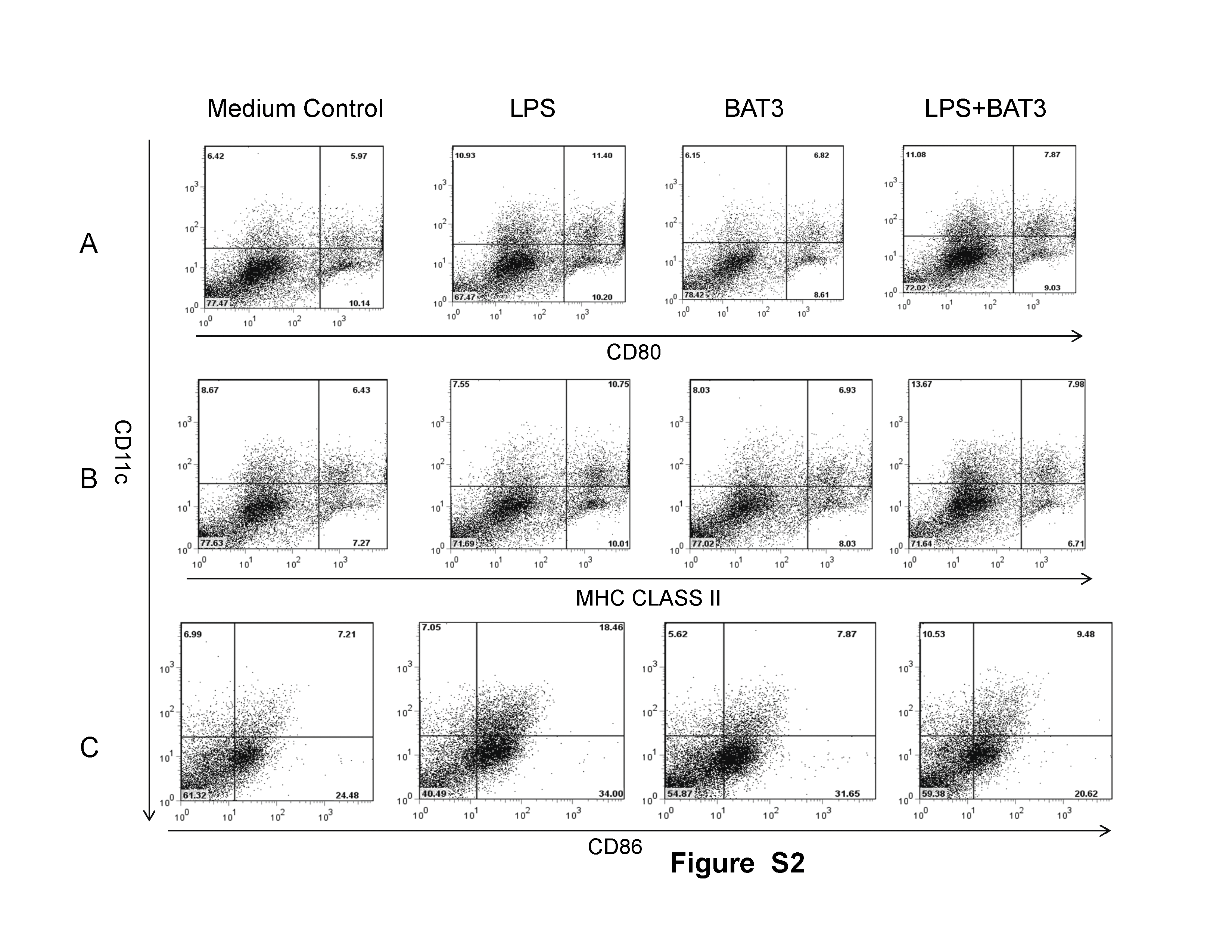

Supplement: Figure S2 — Effects of soluble BAT3 on expression of activation markers on dendritic cells. Murine bone marrow-derived dendritic cells (BMDC) were first stimulated with LPS (100 ng/ml) for 2 hours and then treated with BAT3 (5 µg/ml). The cells were analyzed by flow cytometry for the expression of DC activation markers CD80 (A), MHC class II (B) and CD86 (C) after 48 hours. The cells were gated on CD11c and DC activation markers and dot plots are shown. (TIF) [file pone.0040836.s002.tif]
