# Supplementary material for: BAT3 Regulates Mycobacterium tuberculosis Protein ESAT-6-Mediated Apoptosis of Macrophages
Source: PLoS One. 2012 Jul 13;7(7):e40836. doi: 10.1371/journal.pone.0040836 (PMC3396635; doi:10.1371/journal.pone.0040836)
Supplement: Table S1 — Sequences of the primers used in Real-Time PCR of BAT3 and Molecular Cloning of BAT3 gene. (DOCX) [file pone.0040836.s003.docx]

**Table S1**

---------------------------------------------------------------------------------------------------------------------

Primer Sequences used in Real-Time PCR of BAT3 and Molecular Cloning of BAT3 gene

----------------------------------------------------------------------------------------------------------------------

Real-Time PCR

F- 5’ AACAACAGCAGAAGAAGCCATGTCCCGA 3’

R- 5’ TGCATTGTCTTTCGTCTCTTGGCAGGCA 3’

BAT3-Cloning

F- 5’ CCCAAGCTTCCTTGGTGCCAGGTT 3’

HIND III

R- 5’ CGTGGTACCCAGGAAAATCCGACTT 3’

KPN I
